# A Case of Carbon Monoxide-Induced Delayed Neurological Sequelae Successfully Treated with Hyperbaric Oxygen Therapy, N-Acetylcysteine, and Glucocorticoids: Clinical and Neuroimaging Follow-Up

**DOI:** 10.1155/2019/9360542

**Published:** 2019-05-15

**Authors:** Vincenzo Spina, Francesco Tomaiuolo, Lorenzo Celli, Luca Bonfiglio, Luca Cecchetti, Maria Chiara Carboncini

**Affiliations:** ^1^Department of Translational Research and New Technologies in Medicine and Surgery, University of Pisa, Pisa, Italy; ^2^Auxilium Vitae, Volterra (Pisa), Italy; ^3^Neurology Department, ASLAL, San Giacomo Hospital, Novi Ligure (Alessandria), Italy; ^4^Molecular Mind Lab, IMT School for Advanced Studies Lucca, Lucca, Italy

## Abstract

Carbon monoxide (CO) poisoning is a leading cause of intentional and unintentional poisoning worldwide, associated with mortality and severe morbidity. Some survivors of CO poisoning develop, after a lucid interval, a potentially permanent encephalopathy in the form of cognitive impairment and movement disorders, such as Parkinsonism. One of the most frequent neuroimaging findings is a cerebral white matter damage, but so far its precise cause and specific therapy are still debated. We here report the case of a 33-year-old woman with severe carbon monoxide poisoning who, after a period of lucid interval, presented symptoms of declining motor and cognitive functions. She was treated with 40 sessions of Hyperbaric Oxygen Therapy (HBOT). The therapeutic use of oxygen at supraphysiological pressures might either increase systemic oxidative stress or cause an overproduction of oxygen free radicals as drawbacks. Concurrent use of antioxidants and anti-inflammatory drugs may prevent the side effects of oxygen therapy at supraphysiological pressure due to oxidative stress. For this reason, the patient was also treated with high-dose N-Acetylcysteine and glucocorticoids. Here, we describe the longitudinal monitoring of patient's cognitive abilities and leukoencephalopathy associated with her positive clinical outcome.

## 1. Introduction

At present, carbon monoxide (CO) poisoning remains a leading cause of unintentional poisoning worldwide [1]. In Italy, the estimated incidence is about 6.000 cases per year, resulting in more than 350 deaths per year [2]. The clinical symptoms range from headache and confusion to coma and death, with mortality rates reaching up to 3%.

CO is a nonirritant gas generated during the incomplete combustion of carbon-based compounds. Upon exposure, CO binds to hemoglobin with an affinity known to be 200-300 times greater than oxygen, generating carboxyhemoglobin (HbCO). It decreases both the oxygen-carrying and oxygen-delivery capacity of blood, inducing tissue hypoxia (HbCO theory) [3]. While the hypoxemic hypoxia suggested by the HbCO theory is undoubtedly a key component of CO poisoning mechanisms, it is not enough to account for some of the neuropathological manifestations of CO poisoning. In recent years, researchers have demonstrated CO interaction with soluble guanylate cyclase, ion channels, nitric oxide, nitric oxide synthase, mitochondria, cytochromes, NAPDH oxidase, and xanthine oxidase. Direct effects of CO poisoning on separate organ systems are well explained by these extra-hemoglobin effects, with the most important being the effects upon nitric oxide and on reactive oxygen species (ROS). In addition, a direct effect on cardiac ion channels has been established [4].

A significant number of patients who survive CO poisoning suffer from delayed neurological sequelae (DNS) that typically develop between 27 and 270 days after injury. The DNS reported incidence varies widely from 3 to 40%, because of the lack of established diagnostic criteria. In the classic biphasic presentation, patients recovering from acute CO poisoning manifest a relapse of neurological deficits after a lucid period, including seizures, consciousness disturbances, psychiatric disturbances, movement disorders, and urinary incontinence [5].

The pathophysiology of DNS is not completely understood, though some authors suggested that the typical white matter involvement in CO poisoning reflects a transient intramyelinic edema or inflammation, due to brain lipid peroxidation [6, 7]. Biochemical and immunological studies found that myelin basic protein (MBP) undergoes antigenic alterations after CO-exposure, which suggest that delayed CO-mediated neuropathology is linked to an adaptive immunological response to chemically modified MBP [8]. In the setting of acute CO poisoning, the standard of care is high-flow oxygen, preferentially hyperbaric therapy, and general supportive care [9]. As compared to Normobaric Oxygen Therapy (NBOT), Hyperbaric Oxygen Therapy (HBOT) provides increased dissolved-oxygen content in blood and accelerated elimination of CO. Nonetheless, existing randomized trials do not establish whether the administration of HBOT to patients with CO poisoning reduces the incidence of adverse neurologic outcomes [10].

Currently, in the subacute phase of CO poisoning and/or DNS, there is no standard treatment and clinical case reports do not agree about treatment strategies (e.g., the choice between NBOT and HBOT, the number of sessions) [11–15].

Here, we describe the case of a 33-year-old woman who suffered from CO acute intoxication and was diagnosed with DNS after a period of full recovery (i.e., lucid period). Hyperbaric Oxygen Therapy associated with administration of ROS scavenger was tentatively performed and the positive clinical evolution of the patient was monitored through extensive cognitive testing and radiological assessment.

## 2. Case Presentation

A previously healthy 33-year-old woman was found unconscious at her house. She was exposed to CO from a faulty heater and the exposure time was unknown. She was last heard by her relatives 10 hours earlier, when she referred experiencing nausea, vomiting, and headache.

She was admitted to intensive care unit (ICU) with a Glasgow Coma Scale score of 9/15 (E4-M3-V2). Arterial blood gas sample analysis revealed metabolic acidosis and her HbCO level was 20.4 percent. Serum biochemistry disclosed elevated serum creatinine (1.33 mg/dL) and HS-Troponin T (188 ng/L). Due to her severe neurological symptoms, she was intubated and sedated with propofol. No evidence for alterations of brain tissue was found from either computed tomography (CT) or magnetic resonance imaging (MRI) at this point. HBOT (Drass Galeazzi Underwater Technology, Livorno, Italy) was performed immediately and 24 hours after admission (80 minutes' exposure at 2.5 atmospheres absolute, ATA). After 9 days, the patient showed some spontaneous movement. She gradually regained consciousness and both cardiac and kidney functions improved.

On the 15^th^ day after her ICU admission, she started following commands and was subsequently weaned from the ventilator. She was discharged from the ICU and directed to the rehabilitation unit on the 18^th^ day after admission. At first, the patient showed good adherence to rehabilitation program.

Disability Rating Scale (DRS) [16] measured at this time revealed a score of 8/30, corresponding to moderately severe disability.

However, approximately 40 days after the exposure to CO, she was confused and suffered from slowing of psychomotor functions, impaired short-term memory, and reduced sustained attention. She was no more able to maintain the upright position. The symptoms gradually worsened in few days and she was transferred to our hospital. The patient was hypomimic and presented stereotyped movements, especially with her hands, when she was admitted to the facility. She was not oriented in time and space, had reduced ability to sustain attention, and had optic apraxia as well. Her speech was restricted to short phrases in response to specific requests and she had anterograde memory deficits. She demonstrated very poor awareness of her cognitive deficits. The overall raw score to the Mini-Mental State Examination (MMSE) [17] was 6/30. DRS at this time reached the score of 16/30, corresponding to severe disability.

Motor system examination revealed rigid tetraparesis. There was significant truncal and nuchal extensor hypertonia. She had urinary and bowel incontinence. Examination of the other systems was normal.

Brain MRI performed on day 45 after exposition showed confluent bilateral and symmetric white matter hyperintense areas on T2 and Fluid Attenuated Inversion Recovery (FLAIR) sequences (Figure 2). The hematologic, biochemical, and serological studies as well as HbCO levels were unremarkable. Electroencephalogram showed diffuse slow brain activity. Based on the clinical history and laboratory/imaging findings, the diagnosis of DNS was assumed.

Inpatient rehabilitation program consisted of 2 up to 3 hours of physical and speech therapy per weekday. Therapeutic rehabilitation techniques focused on obtaining a good trunk control in sitting position, readjustment to upright position, muscle strengthening for upper and lower limbs, initiation of gait training, and improvement of orientation and attention.

Rehabilitative treatment was performed concurrently with HBOT and ROS scavenger administration.

HBOT consisted of 40 sessions (two 35 minutes' oxygen breathing cycles with air breathing interval for 5 minutes at 2.5 ATA) with the following sequence: once a day for 20 days, no treatment for 15 days, once a day for 10 days, no treatment for 15 days, and lastly once a day for 10 days again.

ROS scavenger therapy consisted in intravenous high dose N-Acetylcysteine (12 g/die in continuous infusion) and low dosage of oral glucocorticoids (prednisone 25 mg/die).

Since the third session of HBOT, there was a significant improvement in her motor and cognitive skills: the patient became able to stand in upright position with arms on a surface. Her sustained attention began to last much longer and she became oriented in space and time. Language skills improved in both verbal comprehension and production.

After 100 days and 20 sessions of HBOT, she was able to walk for approximately 100 meters using a walker-rollator, however with some gait difficulties. MMSE raw score improved and reached the cut-off level for absence of cognitive impairment (24/30). Despite these improvements, the concurrent analytical neuropsychological evaluation showed performance deficits in several cognitive functions. For this reason, treatment was continued with 20 more sessions of HBOT, rehabilitation, and pharmacological therapy.

The neurological profile gradually improved until the time of discharge (i.e., at the end of the 40 HBOT sessions), when the patient appeared calm, lucid, and oriented and was able to relate to others. Complete motor recovery was observed within 150 days. DRS reached a score of 3/30, disclosing a partial residual disability.

At 18 months of follow-up, neuropsychological tests were all within normal range (see Table 1). The patient was now independent in her activities of daily living and was able to return to her previous job.

**Figure 1:**
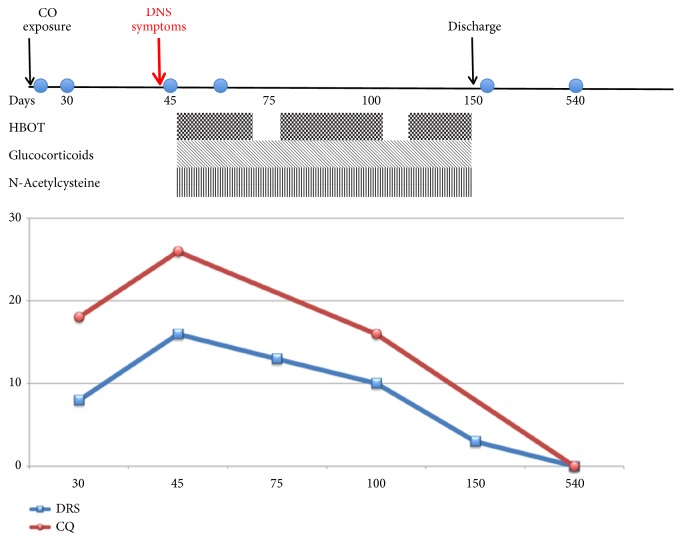
Timeline of clinical course. CO: carbon monoxide. HBOT: Hyperbaric Oxygen Therapy. DRS: Disability Rating Scale (Rappaport et al., 1992). CQ: cognitive quotient. Corresponding MRI scans are indicated as circles in the clinical course.

Figure 1 recapitulates the timeline of patient's clinical evolution.

The neuropsychological evaluation was carried out in multiple sessions, each lasting approximately 1 hour. The patient was sitting and relaxing in a room at a comfortable temperature and was required to perform tests included in the battery named “Esame Neuropsicologico Breve” [18], including a readapted version of the digit span test, trail-making test, word phonemic fluency, immediate and delayed recall of a short story, Brown-Peterson Interference Test, clock drawing test, verbal abstraction test, and Praxia test. To visually synthesize the cognitive skills' follow-up, we created a cognitive quotient (CQ) represented by the ratio between the number of tests under the normal performance range and the maximum obtainable score (two points for each).

**Figure 2:**
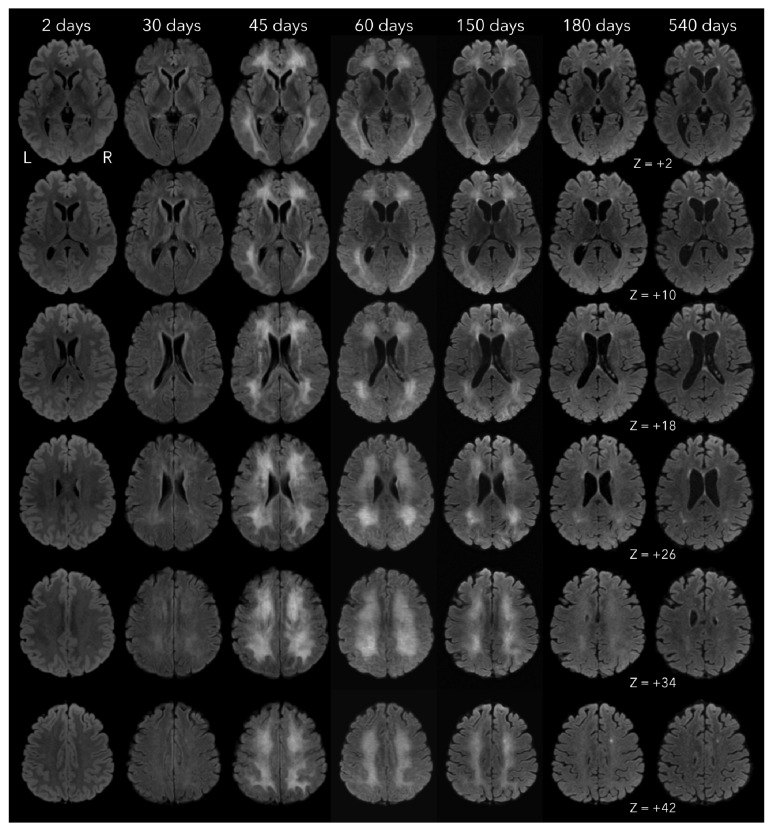
Comparison of MRI scans across different time-points after exposition. 30 and 45 days after initial event, FLAIR sequences show a diffuse and confluent hyperintensity involving predominantly the periventricular and deep white matter. Follow-up MRI scans (60, 150, 180, and 540 days after initial event) show gradual regression of the FLAIR hyperintensity.

MRI scans were performed on the 2^nd^, 30^th^, 45^th^, 60^th^, 150^th^, 180^th^, and 540^th^ days after exposition. MRI images underwent a specific processing pipeline, aimed at improving the inspection and qualitative comparison of patient leukoencephalopathy across time-points. In brief, FLAIR images were first brain-extracted using the FSL brain extraction tool [19]. Afterwards, images were linearly transformed (FSL-flirt; 12 DOF affine registration [20]) to match a high-resolution MNI FLAIR template available at http://brainder.org, using sinc as interpolation method and a final resolution of 1x1x8mm (Hanning window, 7 voxels window width). Finally, to further reduce differences in intensity and contrast among different time-points, preprocessed images were imported in MATLAB v8.5.0, adjusted (imadjust) and filtered using Contrast-limited Adaptive Histogram Equalization algorithm (adapthisteq). The results of this processing pipeline are presented in Figure 2.

## 3. Discussion

We report the successful treatment of a 33-year-old woman suffering from DNS after accidental CO poisoning. Our patient showed a progressive and sustained clinical improvement in motor, cognitive, and behavioral symptoms, accompanied by a reduction on the extent of the white matter abnormalities in the follow-up MRI study.

To our knowledge, there are no reported cases of patients with DNS treated with HBOT and concurrent ROS scavenger therapy. Moreover, our patient was periodically monitored with a comprehensive battery of neuropsychological tests aimed at characterizing her cognitive functions in detail.

Chang et al. [21] used MMSE as an outcome measure after CO poisoning and found a normalization of MMSE scores after the HBOT. In our patient, the normalization of MMSE score long predates the complete resolution of neuropsychological deficits in the different cognitive domains. This suggests the importance of using a battery of neuropsychological tests for the evaluation and follow-up after CO poisoning. There is still limited evidence on the efficacy of HBOT in the subacute phase of CO intoxication and/or DNS. HBOT has complex effects on immunity, oxygen transport, and hemodynamics. Mechanisms of neuroplasticity and cellular repair by HBOT have been suggested in many animal studies [22]. The ability of HBOT to induce angiogenesis was demonstrated in several different preclinical studies [23, 24]. HBOT may also promote neurogenesis of endogenous neural stem cells [25]. Efrati et al. [26] proved that HBOT can lead to significant neurological improvements in poststroke patients even at chronic late stages, implying that neuroplasticity can still be activated long after damage onset.

Furthermore, HBOT has been proposed for the treatment of some autoimmune diseases, given its immunosuppressive effect. HBOT produces changes in lymphocyte redistribution, suppression of antibody production, and suppression of leukocyte and macrophage function [27, 28]. Given the role of adaptive immunological response to chemically modified myelinic basic protein, HBOT could provide some beneficial effects in the treatment of CO-poisoned patients. Thom [29] reported reduced lipid peroxidation with HBOT in rats with CO poisoning. On the other hand, the therapeutic use of oxygen, particularly at supraphysiological pressures, might either increase systemic oxidative stress or cause an overproduction of oxygen free radicals [30]. An imbalance of free radical production has been known to result in deleterious modification of biological macromolecules [31]. ROS are known to mediate O2 toxicity, which for HBOT includes pulmonary injuries and central nervous system effects [32]. In addition, ROS formation may promote the inflammation via endothelial cells, chemotactic signals, and the release of products from the arachidonic acid cascade. In central nervous system, this results in progression of demyelinating lesions [33]. As consequence, the concurrent use of antioxidants and anti-inflammatory drugs may prevent the side effects of oxygen therapy at supraphysiological pressure due to oxidative stress. In this case, we administered low doses of glucocorticoids and high doses of N-Acetylcysteine (NAC). NAC is an antioxidant, used clinically to protect the liver from toxic effects of acetaminophen overdose and as mucolytic drugs. Moreover, in fibrosing alveolitis NAC prevent oxidative damage [34]. The effects of NAC are antioxidant because it acts as a source of cysteine for the synthesis of glutathione [35]. NAC has also weak anti-inflammatory effects, by reducing Nuclear Factor-kB activation [36]. This same mechanism is responsible for powerful anti-inflammatory properties of glucocorticoids [37]. In a clinical study, Xiang [38] reported that the combined application of dexamethasone and HBOT, compared with HBOT alone, resulted in better functional recovery from DNS as assessed by means of MMSE score and National Institutes of Health Stroke Scale (NIHSS) [39] score. Therefore, NAC and glucocorticoids have synergic effects to counteract the supposed physiopathology of DNS and the putative HBOT-induced oxidative stress. Although spontaneous recovery cannot be excluded, our case suggests that a combination of HBOT and antioxidant and anti-inflammatory drugs may produce a positive synergistic effect, which could not be achieved when these therapies are applied independently. Further research is needed to clarify the role of antioxidants in the treatment and the time of complete recovery of DNS after CO poisoning.

## Figures and Tables

**Table 1:** Neuropsychological profile at different times after exposition.

Time after exposition (days)	30	45	100	540	Cut-off
Digit span Test	6	**4**	**4**	6	≥ 5

Immediate recall short story	12	**1**	16	16	≥ 8

Delayed recall short story	13	**3**	15	15	≥ 11

Brown-Peterson Interference Test after 10”	**3**	**0**	6	6	≥ 6

Brown-Peterson Interference Test after 30”	**2**	**0**	6	6	≥ 5

Trail making test -A	**91**	**N. E.**	**97”**	33”	≤ 45”

Trail making test - B	**N.E.**	**N. E.**	**342”**	107”	≤ 140”

Word fluency Phonemic	**3**	**0**	**7**	26	≥ 11

Verbal Abstraction Test	6	4	6	6	≥ 4

Superimposed Silhouettes Test	**20**	**0**	**31**	38	≥ 35

Drawing copy	**1**	**0**	**1**	2	≥ 2

Clock drawing test	**0**	**0**	**0**	9	≥ 8

Praxia Test	**5**	**4**	**5**	6	≥ 6

Token Test	5	5	5	5	≥ 5

Cognitive estimation task	5	**1**	5	5	≥ 4

***Cognitive Quotient***	***18/30***	***26/30***	***16/30***	*0/30*	

N.E. indicates that patient was not able to execute the test.

Bolded scores indicate a test under the normal performance range.

Cognitive Quotient: ratio between the number of tests under the normal performance range and the maximum obtainable score (two points for each).
